# Global health on the front lines: an innovative medical student elective combining education and service during the COVID-19 pandemic

**DOI:** 10.1186/s12909-021-02616-9

**Published:** 2021-03-27

**Authors:** Brandon S. A. Altillo, Megan Gray, Swati B. Avashia, Aliza Norwood, Elizabeth A. Nelson, Clarissa Johnston, Darlene Bhavnani, Hemali Patel, Coburn H. Allen, Sarayu Adeni, Nicholas D. Phelps, Tim Mercer

**Affiliations:** 1 Department of Population Health, The University of Texas at Austin Dell Medical School, 1601 Trinity St., Bldg B., Austin, TX 78712 USA; 2 Department of Pediatrics, The University of Texas at Austin Dell Medical School, Austin, TX USA; 3 Department of Internal Medicine, The University of Texas at Austin Dell Medical School, Austin, TX USA; 4 Department of Medical Education, The University of Texas at Austin Dell Medical School, Austin, TX USA

**Keywords:** COVID-19 pandemic, Online platform, Service learning, Curriculum evaluation

## Abstract

**Background:**

An innovative medical student elective combined student-directed, faculty-supported online learning with COVID-19 response field placements. This study evaluated students’ experience in the course, the curriculum content and format, and its short-term impact on students’ knowledge and attitudes around COVID-19.

**Methods:**

Students responded to discussion board prompts throughout the course and submitted pre−/post-course reflections. Pre−/post-course questionnaires assessed pandemic knowledge and attitudes using 4-point Likert scales. Authors collected aggregate data on enrollment, discussion posts, field placements, and scholarly work resulting from course activities. After the elective, authors conducted a focus group with a convenience sample of 6 participants. Institutional elective evaluation data was included in analysis. Authors analyzed questionnaire data with summary statistics and paired t-tests comparing knowledge and attitudes before and after the elective. Reflection pieces, discussion posts, and focus group data were analyzed using content analysis with a phenomenological approach.

**Results:**

Twenty-seven students enrolled. Each student posted an average of 2.4 original discussion posts and 3.1 responses. Mean knowledge score increased from 43.8 to 60.8% (*p* <  0.001) between pre- and post-course questionnaires. Knowledge self-assessment also increased (2.4 vs. 3.5 on Likert scale, *p* <  0.0001), and students reported increased engagement in the pandemic response (2.7 vs. 3.6, *p* <  0.0001). Students reported increased fluency in discussing the pandemic and increased appreciation for the field of public health. There was no difference in students’ level of anxiety about the pandemic after course participation (3.0 vs. 3.1, *p* = 0.53). Twelve students (44.4%) completed the institutional evaluation. All rated the course “very good” or “excellent.” Students favorably reviewed the field placements, suggested readings, self-directed research, and learning from peers. They suggested more clearly defined expectations and improved balance between volunteer and educational hours.

**Conclusions:**

The elective was well-received by students, achieved stated objectives, and garnered public attention. Course leadership should monitor students’ time commitment closely in service-learning settings to ensure appropriate balance of service and education. Student engagement in a disaster response is insufficient to address anxiety related to the disaster; future course iterations should include a focus on self-care during times of crisis. This educational innovation could serve as a model for medical schools globally.

**Supplementary Information:**

The online version contains supplementary material available at 10.1186/s12909-021-02616-9.

## Background

The COVID-19 pandemic has fundamentally disrupted traditional medical education throughout the world [1]. In order to ensure student safety, prevent further disease transmission, and preserve personal protective equipment (PPE), the Association of American Medical Colleges (AAMC) strongly recommended suspending medical student participation in direct patient care early in the pandemic [2]. Conversion to online or virtual learning can replace some in-person instruction during a pandemic [1, 3, 4]; however, there have been increasing calls for participation of medical students in the COVID-19 pandemic response [5, 6].

The pandemic presents a once-in-a-lifetime disaster response learning opportunity for medical students. Despite a 2003 joint recommendation from the AAMC and the Centers for Disease Control and Prevention [7], few medical schools require disaster training for medical students because of the barriers to incorporating it into a densely packed curriculum [8]. In a survey of 523 medical students, only 26.2% felt adequately prepared for pandemic influenza, yet 93.7% of students in the survey were willing to respond in the event of a pandemic [9]. It is crucial to create safe service-learning opportunities to train future physicians in pandemic response, and the current pandemic has created a window for introducing pandemic response education into the medical student curriculum. Furthermore, medical students are knowledgeable, increasingly experienced, and passionate. In this unprecedented global emergency, balancing medical student learning with clinical and public health service is more important than ever before.

At The University of Texas at Austin Dell Medical School (DMS), we rapidly designed and implemented a medical student elective called “The COVID-19 Pandemic: Global Health on the Front Lines.” Learning objectives were to:

1. Explain clinical and epidemiologic features of SARS-CoV-2 and COVID-19 disease
2. Identify key features of pandemic response at global, national, and local levels
3. Analyze how global pandemics intersect with issues such as health equity, social justice, health system design, healthcare policy, political governance, culture, communications, research, education, and ethics.

The elective had two main components: 1) an online platform with asynchronous modules, readings, discussion boards, and group presentations (10–15 h per week); and 2) pandemic response field placements at a health system or community partner site (25–30 h per week). Most field placements were virtual; none involved patient contact requiring PPE (Table 1).

**Table 1:** Pandemic response field placements in the COVID-19 elective course

Site	Function	N
UTHA^a^ Contact Tracing	• Identify patients with lab-confirmed COVID-19; call all contacts during their infectious period	4
	• Enroll patients with COVID-19 into a home monitoring program	
	• Counsel contacts of positive patients on self-solation or quarantine protocol and refer to testing	
UTHA Home Monitoring Program	• Review literature on home monitoring and design a study to address a knowledge gap	2
	• Perform data collection, analysis, and writing of study findings, and submit for publication	
	• Help develop home-monitoring protocol and other program materials	
UTHA Phone Triage	• Counsel patients calling with symptoms or questions related to COVID-19; refer as needed	4
	• Coordinate referrals to COVID-19 drive-thru testing	
DMS^a^ Community Exchange	• Assist team to vet community needs from local organization and match with creative solutions and/or donations from individuals or businesses	1
	• Review literature on creating improvised PPE for use by first-line responders	
	• Create protocols for infection control while wearing improvised PPE	
DMS Library/ UT Austin COVID-19 Modeling Group	• Collate information on COVID-19 hospitalizations from peer-reviewed literature, state health department sites, news sources, and communication with health leaders in key U.S. cities to inform local modeling of the COVID-19 pandemic	3
CUC^b^ Phone Triage	• Counsel patients calling with symptoms or questions related to COVID-19; provide referrals as needed	3
	• Refer patients to local resources for social needs	
CUC Drive-Thru Testing	• Educate and counsel patients in the drive-thru line on isolation vs quarantine procedures	1
	• Develop educational materials (handouts / pamphlets) on isolation and quarantine for CUC patients	
	• Help manage data capture and data entry during the drive-thru testing process	
LSCC^b^ Phone Triage	• Phone outreach to patients at high risk for complications of COVID-19 and transitioning in-person visits to telemedicine visits	2
	• Counseling patients with questions related to COVID-19	

^a^UTHealth Austin (UTHA) is the clinical practice site associated with The University of Texas at Austin Dell Medical School (DMS)

^b^CommUnityCare (CUC) and Lone Star Circle of Care (LSCC) are two federally qualified health centers in Austin, Texas

The elective was offered to second, third, and fourth-year medical students in three formats: 1) enrichment elective (online learning platform only); 2) two-week clinical elective (online learning platform + field placements); or 3) four-week clinical elective (online learning platform + field placements + capstone project). The online platform was built using Canvas (Instructure, Inc., Salt Lake City, UT) and facilitated by virtual videoconferencing (Zoom Video Communications Inc., San Jose, CA).

Students were divided into subgroups with faculty mentors. For week 1, they were tasked with becoming experts in one of four content areas: COVID-19 clinical and epidemiological characteristics, public health and pandemic response 101, lessons learned from historical pandemics, or public health communications. For week 2, each subgroup analyzed the COVID-19 response in an assigned country or state factoring in political structures, health policy and healthcare delivery systems, culture, socioeconomics, and equity (Table 2). Our librarian provided an overview of COVID-19 resources and search strategies [10, 11].

**Table 2:** Online learning platform content areas

Group	Week 1 – Fundamentals of COVID-19 and Pandemic Response	Week 2 – Country/State Case Studies
1	COVID-19 Clinical and Epidemiology	
1A	Clinical Features of COVID-19	Kenya
1B	Epidemiology of SARS-CoV-2 and COVID-19	Singapore
2	Public Health and Pandemic Response 101	
2A	Fundamental Principles of Public Health	Austin, Texas, United States
2B	Fundamental Principles of Pandemic Response	South Korea
3	Pandemic Response: Lessons Learned from History	
3A	1918 Influenza, HIV, SARS	Italy
3B	MERS, H1N1 Influenza, Ebola	China
4	Public Health Information and Communications	
4A	Public Health and Group Messaging	Mexico
4B	Interpersonal Communication	Seattle, Washington, United States

Mimicking the workflow of pandemic response teams, groups held mandatory virtual “daily huddles” to discuss insights and share work. Each subgroup posted meeting minutes for accountability and met virtually with their faculty mentors for mid-week checkpoints. To foster dialogue and interactive learning, faculty mentors posed discussion board questions (Additional file 1); students were required one original post and one response. At the end of each week, subgroups gave a 15- to 20-min presentation on their content area via videoconference and provided an annotated bibliography of key articles. To ensure tangible benefit to the community beyond the field placements, each subgroup completed a community engagement public health education project by the end of week 2, creating a deliverable for a specific target audience while applying new skills in health information messaging and advocacy.

In addition to field placements, students in the four-week track were paired with a faculty mentor to complete a capstone project. The capstone prompt was to critically examine the pandemic in light of health equity, health system design and policy, research and education, culture, and ethics. Faculty reviewed student proposals in the second week, provided feedback and suggested mentors for academic, communications, humanities, or creative products.

The purpose of this study was to evaluate the students’ experience in the course, the curriculum content and teaching format, and the short-term impact of the course on students’ knowledge and attitudes around the COVID-19 pandemic. In evaluating the students’ experience and the curriculum, our goal was to evaluate the course’s intrinsic merit, while the evaluation of short-term impact on knowledge and attitudes was an assessment of the extrinsic value of the course.

## Methods

This was a curriculum evaluation study including both quantitative and qualitative data. The University of Texas at Austin Institutional Review Board (IRB) assigned the study exempt status. All methods were carried out in accordance with relevant guidelines and regulations.

### Participants and research design

All participating medical students were included in the evaluation. Basic aggregate information collected included the students enrolled per academic year, the number of original discussion posts and responses, the number of student field placements, and descriptions of scholarly work resulting from course activities.

Students completed an anonymous pre-course questionnaire (Additional file 2) to assess their baseline pandemic knowledge, prior public health experience, and to-date involvement in and concern about the COVID-19 pandemic. They also submitted an initial reflection piece on personal impacts of the pandemic. At the end of the two-week course, all students completed an anonymous post-course questionnaire (Additional file 2) to reassess knowledge and attitudes. They also submitted a final written reflection on their course experience and health equity or systems issues exacerbated by the pandemic. Data from the standard institutional course evaluation form (Additional file 3) was also used.

At the conclusion of the elective, a convenience sample of six students participated in a virtual focus group to share their course experience and its perceived value in their careers (focus group discussion guide is shown in Additional file 4). Students were recruited from both two- and four-week cohorts; all academic years were represented.

### Data collection procedure

The questionnaires, reflection pieces, discussion boards, and capstone projects were collected as part of educational evaluation, thus no consent was requested as per IRB guidelines for exempt studies. Questionnaire data was anonymous and analyzed in aggregate, so no identifying information was included. Although reflection pieces and discussion board posts were not submitted anonymously, the data was analyzed and de-identified. Participants in the focus group were informed verbally at the outset that participation was optional and would not affect their standing in the course, and verbal informed consent was obtained from all participants. The focus group was audio-recorded and transcribed using a commercial transcription service (Trint, Ltd., London, UK); transcripts were de-identified.

### Data analysis

Questionnaire data was analyzed quantitatively with simple summary statistics and paired t-tests to compare knowledge and attitudes before and after the elective experience. Though our sample size was relatively small and therefore risked violating the normality assumption for parametric testing, consensus among statisticians is that parametric tests are relatively resilient to non-normality except when deviations from normality are severe [12]. We calculated that our sample size of 27 students gave us ≥80% power to detect a mean difference of 0.5 between pre- and post-course questionnaires. Reflection pieces, discussion board posts, and focus group data were analyzed using qualitative content analysis with a phenomenological approach [13].

We drew from Creswell’s questions regarding the group’s experiences of the phenomenon (living through the start of a global pandemic) and what contexts influenced their experiences [13]. Using Nvivo software (QSR International, Melbourne, Australia), student reflections and focus groups were mined for clusters of meaning. Three study investigators each coded two-thirds of the qualitative data, so that all data was coded twice. These investigators then reviewed the codes together and created a consensus coding framework. These codes coalesced into broader themes based on frequency and distribution. Representative quotes were extracted for each theme. This final list of themes and representative quotes was reviewed by all investigators and any disagreements were resolved by consensus. The themes were reflective of this lived experience and allowed us to better characterize and understand the phenomenon.

## Results

### Process metrics

Table 3 describes the 27 students enrolled in the course; two additional students participated in a service-learning experience organized by the course but not in the didactic component. Seven students enrolled in the enrichment elective only, eight enrolled in the two-week elective, and 12 enrolled in the four-week elective.

**Table 3:** Summary demographic data of participants in the COVID-19 elective course

	Total [N (%)]
**Total enrollment**	**29 (100.0)**
2-week clinical elective	8 (27.6)
4-week clinical elective	12 (41.4)
Enrichment only	7 (24.1)
Service-learning only	2 (6.9)
**Year**	
MS2	2 (6.9)
MS3	20 (69.0)
MS4	7 (24.1)
**Sex**	
Female	19 (65.5)
Male	10 (34.5)
**Race/Ethnicity**	
Asian	8 (27.6)
Black	2 (6.9)
Latinx	1 (3.4)
White	18 (62.1)
**Prior public health experience**^**a**^	
Degree or coursework	8 (27.6)
Practical/career experience	6 (20.7)
None	14 (48.3)
No response	3 (10.3)

^a^Does not total 100%; some reported both degree/ coursework and practical/career experience

During the course, 67 original discussion posts were generated, 65 from students and two from faculty. There were 122 responses to these posts, 84 from students and 38 from faculty. There was an average of 2.4 original posts and 3.1 responses per student. The posts ranged widely in topic. Students discussed SARS-CoV-2’s clinical and epidemiological characteristics, demonstrated understanding of basic public health concepts, and proposed public health actions. They critically evaluated the US pandemic response, drawing comparisons with other epidemics and with other countries’ pandemic response measures. They discussed the influence of cultural and public opinion on pandemic response and described public health communication principles. Finally, they discussed societal impacts and systemic implications of the pandemic, including disparities in impact among vulnerable populations, stigma towards Asian-Americans, economic impacts, and healthcare system deficiencies. Table 4 lists common themes and representative quotes from the discussion board posts, pre−/post-course reflections and the focus group. The reflections and focus group discussion revealed the students’ experience with COVID-19 more broadly, outside of the course context; these findings will be reported separately.

**Table 4:** Common themes and representative quotes from student responses^a^ in the COVID-19 elective course

Theme	Subtheme	Representative Quote
Background knowledge	Public health concepts	“I was shocked to learn that 80% of viral infections and 50% of bacterial infections are zoonotic. […] viruses get passed back and forth between different animal species all the time, which is what leads to their incredible diversity.”
		“I’d heard the flatten the curve term and it made sense theoretically, but then sort of seeing some of the projections that came out of Meyer’s lab about […] here’s our capacity in beds and ventilators, etc. And then here’s how if we don’t […] really aggressively flatten the curve, how we’re gonna fly past capacity. That was overwhelming.”^a^
	COVID-19 clinical and epidemiological knowledge	“The exact combination of SARS-CoV-2’s relatively low case fatality rate (CFR) overall, R0, and possibility of transmission from asymptomatic patients lead [s] to the issues that we are experiencing with COVID-19.”
Public health response	Cultural and social factors in response	“If we consider East Asian countries to be on one end of the scale of individualism/collectivism, and America is on the end, I wonder where a lot of the European countries fall on that scale. […] in France, for example, solidarity is a strong value […] However, their governments are not as authoritarian as the Chinese government, and the European view on civil liberties probably veers closer to the American perspective. […] if the French government imposed such draconian measures on their population, it would not be taken kindly.”
		“The online course has allowed me to explore the responses from different countries and compare them to what is happening here to see what is being done well and what is being done poorly. For example, what is being done in the Asian countries such as China, Singapore and South Korea would be much harder to implement here given the differences in culture.”^b^
	Ethical dilemmas	“As healthcare providers, we often straddle the line between patient autonomy and paternalism […] With the health and well-being of many other people at stake, I wonder if, during this critical period, […] autonomy should take a backseat to the advice of medical professionals and public health officials.”
	Comparisons with other viruses and pandemics	“SARS and MERS taught us that hospital systems need to have standardized protocols for dealing with pandemics, and they need those protocols to be set in place before the pandemic hits.”
		“The public’s response in China and South Korea, their willingness to engage in government orders may also have to do with fear of the outcomes of the previous pandemic’s experienced in these countries.”^b^
	Comparisons among countries	“[The village of Vo in] Italy also has what I think is one of the most stunning examples for stopping the epidemic in its tracks. […] Without finding and isolating the asymptomatic cases, there’s no way such a profound decrease in infections could have happened. […] South Korea and Taiwan have shown success with similar strategies. I fear it is too late to implement something of that scale here in the US.”
		“After spending the second week of the elective studying Kenya’s response and preparations, I continue to draw correlations to rural America.”^b^
	Criticism of response	“Prompt response requires resources and a governmental department [not] strapped by low human and financial resources. […] it seems that we have the expertise and structures to have the correct system [s …]. However, [we allow] other interests to get in the way of optimal preparedness and response.”
		“I oft find myself reflecting on what could have been if the US prioritized public health and a social safety net like many other developed countries. It’s highly unfortunate that we as a society have come so obsessed with increasing margins and profits that we overlooked critical health infrastructure such as the National Stockpile and PPE. Now, we must pay the cost in human life and in productivity.”^b^
	Public health solutions	“Early testing, aggressive contact tracing, and quick isolation is our best line of defense […]. I don’t think that Austin as a city is there yet, but […] there are pockets of our community fighting hard for this.”
	Public health communication	“It is important to have an idea of what the audience already knows or believes […] add to the audience’s knowledge and correct any misconceptions […]. The Health Belief Model states that people will take into account the perceived severity and risk of a health event, […] barriers to, benefits, and risks of action.”
		“more confident in my ability to access helpful resources.[…] I was in [the group] that worked […] on interpersonal communication, they created this really comprehensive, really good blog post. […] if I was in that situation, I would just immediately go to their blog post and read some responses guiding me on that.”^a^
		“I realized that health literacy was an issue and no one was sure if CDC guidance applied to small, rural towns.”^b^
Community impact	Public opinion and behavior	“I think social opinion is in favor of extreme measures to contain the pandemic. However, if the measures are good then we will never know how bad the pandemic could have been, leading to increased speculation as to whether the interventions were appropriate. Already, there are individuals calling for the reopening of the economy, stating that the ‘cure shouldn’t be worse than the disease,’ which seems to downplay the rhetoric of how many people could die or be hospitalized due to this disease. I’m curious to see how public opinion evolves as social distancing, quarantine and isolation continue past novelty.”
		“Our family members [in China] are worried about us because they do not believe the US is responding adequately to the crisis, and because they have heard instances of people of Asian descent unfortunately being the target of discrimination and assault due to scapegoating and xenophobia.”
	Disparities in impact	“[…] despite the great strides we have made in public health, medicine, and sanitation, many of the structural factors that aided in the spread of influenza still exist today. There are even greater economic disparities, with the lower class living in areas of the city with decreased access to healthcare, poorer social support, and less resources. Practices of social isolation are extremely difficult when they mean that people living paycheck to paycheck will lose their homes. How does someone without a car get around if they cannot use public transportation? And shelter-at-home can be difficult if you do not have access to any kind of stable housing.”
		“the impacts that we’re seeing on our most vulnerable communities and how any crisis […]illuminates, […] those inequities arising […] around race and ethnic lines or around being undocumented in this country.”^a^
		“There is now a growing interest in inequality of cases distribution—disease prevalence overlapping areas with a higher proportion of service workers and lower cost-housing, predominately black communities. The pandemic is revealing much about the US—Inequalities, systemic racism; wage and worker conditions; differences in education and health literacy.”^b^
Systemic implications	Economic impact	“The mitigation conversations seem to be playing with this balance of how bad is the virus versus [the] recession. Some [sources] report that not only are the mitigation efforts necessary […] but also that a reopening of the economy at this point would be generally detrimental. I see a lot of talk in politics about ‘the cure being worse than the disease,’ but that talk sounds like profit vs. lives, which is not a responsible conversation to have. However, the argument can be made that, for however much unemployment rises, suicides and/or all cause death rises, so that would complicate the argument for listing restrictions.”
	Political and policy implications	“This discussion over protection for financially vulnerable families in Texas is one of policy. Unlike other wealthy, industrialized nations, the United States does not mandate paid sick leave or universal healthcare. In the face of this pandemic, public health experts and policy makers are being asked to create legislat [ion] that allows for a safety net for these populations. Since any legislat [ion] is slow-moving […], public health officials are scrambling to […] meet needs on a daily basis by tapping into philanthropic and non-profit organizations as they wait for federal ordinances […]. I’d be curious how other countries with paid leave and universal healthcare are faring in comparison.”
		“This pandemic does raise novel […] and interesting questions about how to evaluate government intervention and balances of power (both between branches of the federal government and between the federal and state governments).”^b^
	Health system implications	“I would like to think that this public health crisis will be an opportunity for all of America to critically reexamine our existing healthcare system. It is making people aware more than ever before how broken it is and how critically necessary it is to fix it so that we can have a healthcare system that works for us, not against us.”
		“Fundamentally, I think medicine will always be the same. But I think this is going to cause us to question a lot of things. Look back at a lot of our policies and practices, the way we do things. And I think a lot of things are going to change by the time we actually get out into the force.”^a^

^a^The majority of highlighted responses were from discussion board posts; those from focus groups (a) and reflection papers (b) have been noted accordingly

The first week’s presentations lasted 191 min and were attended by 26 of the 27 students and 14 faculty/staff. The second week’s presentations lasted 211 min and were attended by all 27 students, one additional non-enrolled student, and 14 faculty/staff. After gaining student permission, the video-recorded presentations along with copies of the presentation files and annotated bibliographies were posted on the DMS intranet site for broader consumption.

Table 5 describes the students’ community-engaged public health education projects and capstone projects. The eight capstone projects completed ranged from op-eds to pilot data submitted for publication.

**Table 5:** Community-engaged public health education projects and capstone projects produced by students in the COVID-19 elective

Project Type	Project Description
**Community-engaged public health education projects**	
Video series	3 short tutorials on the basics of COVID-19, isolation, and quarantine aimed at community health center staff (clerks, medical assistants, nurses) and the general public
Policy brief	Information for local policymakers and health system leaders on creating disease-specific hospitals in response to an emerging infectious disease
Policy brief	Information for local policymakers and housing agencies on leveraging the crisis as an opportunity to address homelessness in Austin
Slide deck and flyer	Information for local public health leaders on designing effective messaging for Millennials on social distancing; sample flyer demonstrating effective messaging techniques
**Capstone projects**	
Op-ed	Discussion of higher risk to women of interpersonal violence due to the shelter-in-place order
Op-ed	Discussion of the pandemic’s impact on the practice of primary care and chronic disease management, and opportunities for new care delivery models
Poem	Reflection on the impact of the COVID-19 pandemic on our daily lives and routines
Poetry series	Collection of sestinas using words submitted from medical students and faculty describing personal experiences with the pandemic
Community impact project	Creation of a new volunteer communication and onboarding strategy using a human-centered design approach for a local non-profit to provide free food delivery to vulnerable families during the pandemic
Epidemiologic report	Manuscript describing the role of asymptomatic spread in a locally identified cluster of students
Epidemiologic report	Pilot study of an active surveillance testing strategy among asymptomatic healthcare workers in Austin
Community impact and research project	Creation of database of local food insecurity resources for distribution to families in pediatric clinics; detailed report on how COVID-19 has exacerbated food insecurity in Austin, with a focus on families and children

### Impact on students

Students’ reported reasons for taking the course fell into five themes, in descending order of prevalence:

1. involvement in the pandemic response and service to the local community,
2. general knowledge or understanding about the pandemic,
3. professional development – preparation for residency or for their future careers,
4. ability to fluently discuss the pandemic and counsel others (including colleagues, patients, friends, and family), and
5. need for elective credit.

Questionnaire results are summarized in Table 6. Mean pre-test knowledge assessment score was 43.8%, while mean post-test score was 60.8% (*p* <  0.001). Mean subjective self-assessment of knowledge about COVID-19 and pandemic response, using a 4-point Likert scale, increased from 2.4 before the course to 3.5 after course completion (*p* <  0.0001). Students reported increased confidence in their ability to lead a conversation about limited PPE with clinical staff (2.2 before course to 3.3 after course, *p* <  0.0001), discuss limited ventilators with patients’ families (2.1 vs. 3.1, *p* <  0.0001), and address fear and anger with patients and families in the setting of COVID-19 infection (2.4 vs. 3.3, *p* <  0.0001).

**Table 6:** Summarized pre−/post-course questionnaire data from the COVID-19 elective course

Question	Pre-course	Post-course	***p***-value^a^
What is your level of knowledge about COVID-19 and pandemic response BEFORE/AFTER participating in the activity?^b^	2.4	3.5	< 0.001
I feel confident in my ability to lead a conversation about limited PPE resources with clinical staff.^c^	2.2	3.3	< 0.0001
I feel confident in my ability to lead a conversation about limited ventilators with patients’ families.^c^	2.1	3.1	< 0.0001
I feel confident in my ability to address fear and anger with patients and families in the setting of COVID-19 infection.^c^	2.4	3.3	< 0.0001
I have been meaningfully engaged in the local pandemic response.^c^	2.7	3.6	< 0.0001
The service-learning activities in the course contributed to my learning about pandemic response.^c^	n/a	3.6	n/a
What is your current level of anxiety or concern about the pandemic?^b^	3.0	3.1	0.53

^a^Paired two-tailed t-test

^b^4-point Likert scale: None, minimal, moderate, high

^c^4-point Likert scale: Strongly disagree, somewhat disagree, somewhat agree, strongly agree

In the post-course questionnaire short-answer responses, post-course reflections, and focus group, students reported increased appreciation for the field of public health as a result of the elective. Representative quotes include the following:

*The public health perspective utilized in this course highlighted that this disease can only be tackled with societal engagement. Medical care [is] critical to saving individual lives, but the public health measures, the research with knowledge shared across nation [al] lines is what will save the world.* (MS3, post-course reflection)

*[ … the elective] forced me to think deeper about the public response that was happening around me. When there was a national state of emergency, I accepted it immediately at the time. But this assignment forced me to ask myself, what does this really mean? Why is this possible? What does this unlock? Can they declare something more extensive in the future? The topic also had me question how the local public health worked, who made the local rules, and if those could be trumped by state or federal rules. I read an extensive amount about all of these topics [ … ].* (MS4, post-course reflection)

Several students expressed that the elective increased their understanding of how public health is operationalized and how dynamic it is in practice. Representative quotes include the following:

*It has been so eye-opening to see how much time must be put to ensure the safety of so many. [ … ] when I imagine [d] emergency responses, I imagined teams of professionals working in synch to battle this pandemic, when the reality is so much more chaotic, fluid, and disjointed. I have been fortunate to have this experience to see how things operate without a plan in place, without a formula to follow. So much of medical school is prescribed, if you follow instructions you will do well. Here, I got to see what happens when no instructions exist.* (MS3, post-course reflection)

*[ … ] it continues to be an extremely rewarding experience to see the way that [public health] transpires on the front-lines, when the threat is present and advancing, and the goals (and the means by which they are achieved) are continually evolving.* (MS3, post-course reflection)

Many students reported that the elective caused them to consider integrating public health into their future careers. One student wrote that the elective “[ …] encouraged me to further evaluate [ …] how I can make public health a priority during my future career” (MS2, post-course questionnaire); another stated that as a result of the elective, “I certainly respect the field more and feel even more inclined to seek out getting an MPH in the future [ …]” (MS4, post-course questionnaire). However, at least one student reported that the elective reaffirmed their decision *not* to pursue a career with a public health orientation: “This elective has confirmed that public health is incredibly complicated [ … which] has affirmed my desire to not pursue public health as a significant part of my future career” (MS3, post-course questionnaire).

Students reported that the course did achieve the goal of improving their fluency in discussing the pandemic with others and advising their loved ones. One student wrote:

*[ …] the elective [ …] improv [ed] my ability to speak with peers and others with whom I am close about the pandemic. This includes friends of mine who [ …] were careless with their actions, refusing to abide by social distancing recommendations. Inevitably, several of these friends [ …] tested positive for coronavirus. I was able to coach them throughout their illness course on ways to mend and next steps to take [ … ]. My community involvement also extends to my parents, who have relied on me for much of the news and recommendations regarding the pandemic.* (MS3, post-course reflection)

Questionnaire data showed no significant difference between students’ level of anxiety about the pandemic before and after course participation (3.0 before course to 3.1 after course, *p* = 0.53). Qualitative data from the post-course questionnaire, post-course reflections, and focus group corroborate this finding; there were an approximately equal number of references to the elective decreasing and increasing students’ sense of anxiety around the pandemic. Those who reported that the elective decreased their sense of anxiety often reported that the course gave them a sense of purpose or agency, which counteracted their sense of uncertainty or powerlessness. One student wrote:

*At a personal level, focusing on the elective allowed me to take a break from the anxiety of mindless scrolling through the news on my own. Really digging into the material for a purpose of teaching others allowed me to understand it, have a purpose in seeking information, prepared me to talk about it reasonably, and gave me insight to question each piece of information I was reading.* (MS3, post-course reflection)

Those who reported increased anxiety because of the course often discussed that their increased knowledge made them even more concerned about the implications of COVID-19. One such student wrote, “I wish I could say this elective has made me feel better. While I know much more about it [now] than I did before, I think the more I learn, [ …] the more I see the reality of what is happening with this epidemic” (MS3, post-course reflection). A third, smaller group reported that the course had no impact on their sense of anxiety. One student wrote, “If I’m being honest, the course hasn’t changed anything. I think I’m just numb to the constant onslaught of information and fear at this point” (MS3, post-course questionnaire).

### Course evaluation

The standard institutional elective evaluation was completed by 12 of the 27 enrolled students (44.4%). All respondents rated the course overall as “very good” (*N* = 5, 41.7%) or “excellent” (*N* = 7, 58.3%). All respondents either agreed (*N* = 5, 41.7%) or strongly agreed (*N* = 7, 58.3%) that the elective activities helped them achieve the stated learning objectives. Nearly all respondents (*N* = 11, 91.7%) strongly agreed that the elective promoted skills for self-directed/lifelong learning. Nearly all respondents either agreed (*N* = 6, 50%) or strongly agreed (*N* = 5, 41.7%) that they felt better prepared for residency based on their work in the elective. Nearly all respondents (*N* = 11, 91.7%) reported that the clinical responsibility in the course was appropriate for their level of training.

In response to open-ended questions, the most favorably reviewed aspects of the course were the field placements, the opportunity to learn from peers and work as teams, self-directed research, and the resources and readings suggested by course faculty. Many students commented that the course was well-organized despite its rapid inception. Opportunities for course improvement identified by students were more clearly defined expectations, especially in the field placements, and improved balance between volunteer and educational hours. Several students felt that the overall time commitment was more than anticipated and that either volunteer hours or didactic expectations should be reduced.

Overwhelmingly, the pandemic response field placements were reviewed favorably in post-course reflections. Students reported that participating in the local pandemic response was valuable because it provided them the opportunity to serve their community, gave them a sense of control, and allowed them “patient” contact when removed from the clinical setting. Representative quotes include the following:

*It felt good to be able to make a useful contribution in a world in which people seem to be increasingly powerless. I was impressed at how quickly teams were pulled together, how motivated everyone was to help, and how flexible everyone was in adapting to arising needs.* (MS4, post-course reflection)

*I came home exhausted, but grateful to play a small part in the effort Austin was making to flatten the curve locally. In a way, working with the contact tracing team allowed me some control over the chaos that is reigning all over the country right now. While facing the unknown of lockdowns, ventilator shortages, and sorrow that can come with losing loved ones, this elective and my time on the ground has given me purpose and allowed me not only to learn, but to grow into a more public health minded future physician.* (MS3, post-course reflection)

*I didn’t realize how much I had missed patient care until I started taking phone calls, answering questions and notifying people of their results. It’s one of the best parts of this elective. Filling out the screening form is reminiscent of taking a history – eliciting details about symptoms, possible exposures, etc. [ …] During this time when medical students aren’t allowed in clinical settings, this role feels like a close second.* (MS3, post-course reflection)

Students reported significantly increased engagement in the pandemic response after the course compared to before (2.7 before course to 3.6 after course, *p* <  0.0001). By the end of the course, all surveyed students either somewhat (44.4%) or strongly (55.6%) agreed that they had been meaningfully engaged in the pandemic response, and all either somewhat (37.0%) or strongly (63.0%) agreed that the service-learning activities contributed to their learning about pandemic response.

## Discussion

In this paper, we describe the rapid conception, implementation, and evaluation of an innovative COVID-19 medical student elective. We evaluated the students’ experience in the course, the curriculum content and teaching format, and the short-term impact of the course on students’ knowledge and attitudes around the COVID-19 pandemic. Students removed from clinical rotations were hungry for knowledge about COVID-19 and eager to participate in the pandemic response. Tremendous flexibility and creativity were required from faculty and students to develop a timely elective about an evolving pandemic. Group-based, self-directed learning with faculty support, frequent virtual meetings and active discussion boards, paired with service, created a rich learning environment. Other recently published reports on medical student involvement in the pandemic response focused on their capacity for service [14, 15], while our course combined service-learning with a didactic experience to foster foundational public health knowledge.

Process outcomes showcase high course engagement, with participation of nearly half of all third-year students. While students were only asked to submit one original discussion board post and one response, the observed number of posts demonstrates active student engagement beyond course requirements. Course participation extended beyond enrolled students to the entire DMS community, as demonstrated by non-teaching faculty attending the weekly virtual student presentations. The elective has been highlighted in university and public media [16, 17], underscoring the important contribution of academic centers to community knowledge during public health crises.

The course succeeded in improving student knowledge around various COVID-19 topics. However, the improvement seen between pre- and post-course questionnaire knowledge was only modest. Because the course content was largely student-led, coverage of topics included in the questionnaire may have varied between different student groups. Future course iterations will revise the knowledge evaluation to cover more general public health and basic virology concepts and improve alignment between course activities and desired knowledge outcomes. Students did report subjective improvement in their knowledge and confidence discussing COVID-19-related topics, which addresses one of the principal reasons that students reported taking the course: to improve their fluency discussing the pandemic with others. Students were overwhelmingly positive about the public health messaging and information material; this may be a key area for future critiques and practice, particularly for a non-medical audience.

Students evaluated the course favorably in our course evaluation, with a smaller group completing the institutional evaluation. They particularly enjoyed the field placements, and many reported that active participation in the pandemic response was a therapeutic activity in coping with the uncertainty of this tumultuous time. There were reports from students who felt that the time commitment for their placement was more than expected, or that their clinical level of responsibility was above their level of training. Course faculty recognized these issues during the course through real-time feedback and addressed these concerns by discussing with field placement leaders. Future course iterations will prioritize the right balance between education and service, engaging public health experts in teaching directly during field placements and including time for debrief and reflection.

Despite reports from some that participation in the pandemic response helped students manage their anxiety, there was no significant net impact on anxiety in survey data. Given the rapidly evolving nature of the pandemic, the impact of the course on student anxiety may have been confounded by increasing local impact of the virus and/or by advances in our knowledge about the virus and its spread during the duration of the course. Students did report that knowledge was both empowering and frightening, and we see that duality in the data. Although ameliorating student anxiety was not a stated purpose of creating the course, in the future, course faculty may incorporate recommendations or reflections on self-care and personal wellness in course content. Compared to traditional evaluation metrics, the intrinsic value of activities to reduce anxiety and improve personal and professional functioning during the ongoing COVID-19 pandemic should be of high interest to other academic institutions.

This study has several limitations. The pre- and post-course questionnaire questions were not validated, and the response rate to the institutional course evaluation was suboptimal. The focus group used a convenience sample of students, which may not have been representative of all student experiences in the course. The evaluation was done in real-time and cannot assess longer-term impacts of the course on student knowledge and attitudes. At the close of the course, the pandemic had affected students for under 2 months; at the time of writing we are now facing 4 months, and student responses may well have changed. Balancing these limitations is the robust combination of quantitative and qualitative data for a comprehensive, multifaceted evaluation of the course.

Future directions include a second, smaller-scale evaluation of the long-term impact of the course on students after 1 year. We also hope to offer a significantly updated course that reflects our advancing understanding of COVID-19 and its impact on global populations. The lessons learned from the evaluation will inform adjustments in the structure of the course as described above.

## Conclusion

We hope this course description and evaluation data can be used to implement similar courses at other institutions. Though the COVID-19 pandemic has resulted in significant disruption and tragedy worldwide, guided student engagement in the pandemic response represents a unique and impactful hands-on learning opportunity for medical educators and students. It is imperative that academic medical institutions take this opportunity to identify effective ways to prepare future physicians to advocate for robust public health responses in pandemics [18]. As one MS3 student noted in his post-course reflection: “[ …] hopefully we have learned that we need to invest in preparation—resources, systems, policies, education, medicine, and public health. I hope we also see this as an opportunity to improve upon what the pandemic has revealed about our society.”

## Supplementary Information

**Additional file 1.** Discussion board prompts.

**Additional file 2.** Pre-post questionnaires.

**Additional file 3.** Institutional elective evaluation form.

**Additional file 4.** Focus group consent script and discussion guide.

## Data Availability

The datasets used and/or analysed during the current study are available from the corresponding author on reasonable request.
