# Supplementary material for: Global health on the front lines: an innovative medical student elective combining education and service during the COVID-19 pandemic
Source: BMC Med Educ. 2021 Mar 27;21:186. doi: 10.1186/s12909-021-02616-9 (PMC8003893; doi:10.1186/s12909-021-02616-9)
Supplement: Supplementary file 1 — Additional file 1. Discussion board prompts. [file 12909_2021_2616_MOESM1_ESM.docx]

**Global health on the front lines: An innovative medical student elective combining education and service during the COVID-19 pandemic**

**Authors and Affiliations:** Brandon S. A. Altillo, MD, MPH^1,2,3^, Megan Gray, MD, MPH^1,2^, Swati B. Avashia, MD^1,2,3^, Aliza Norwood, MD^1,3^, Elizabeth A. Nelson, MD^3,4^, Clarissa Johnston, MD^3,4^, Darlene Bhavnani, PhD, MPH^1^, Hemali Patel, MD^3^, Coburn H. Allen, MD^2^, Sarayu Adeni, MPA-DP^1^, Nicholas D. Phelps, PhD^1^, and Tim Mercer, MD, MPH^1,3^

^1^Department of Population Health, The University of Texas at Austin Dell Medical School, Austin, Texas, USA

^2^Department of Pediatrics, The University of Texas at Austin Dell Medical School, Austin, Texas, USA

^3^Department of Internal Medicine, The University of Texas at Austin Dell Medical School, Austin, Texas, USA

^4^Department of Medical Education, The University of Texas at Austin Dell Medical School, Austin, Texas, USA

**Corresponding Author:**

Dr. Tim Mercer, Department of Population Health, The University of Texas at Austin Dell Medical School, 1601 Trinity St., Bldg B., Austin, TX 78712, USA; telephone: 512-495-5393; email: [tim.mercer@austin.utexas.edu](mailto:tim.mercer@austin.utexas.edu).

**Additional File 1: Discussion Board Prompts**

Week 1, Group 1: COVID-19 Clinical Knowledge and Epidemiology

- Compare and contrast the clinical presentation of SARS, COVID-19, a viral URI (“common cold”), and Influenza. What are the key differentiating factors, clinically? How does this affect treatment decisions? How do the similarities drive testing strategies? How do these similarities affect public health messaging? How should patients and families and the public be educated on this?
- Discuss the concepts of isolation, social distancing and quarantine. Given what you know about the incubation period and duration of viral shedding, what time frame should be enacted for each of these measures in order to stop spread of infection? What other factors should be considered when making these decisions, either at a personal level or population level? What subpopulations need special consideration and how should policies and protocols related to these concepts be adapted to different sub-populations?
- Discuss the epidemiology and factors associated with the transmission dynamics of SARS-CoV2. What makes this disease so highly contagious? Discuss the push factors involved in zoonotic transmission of respiratory viruses, and what can be done to reduce the risk of future pandemics of zoonotic origin? Discuss implications for health care workers and what we need to know about transmission dynamics to enact appropriate policies for health care workers. What implications does this have for the general public, in terms of how we educate the public and how we enact policies to prevent the spread of the pandemic?

Week 1, Group 2: Public Health and Pandemic Response

- What was your initial response to the local public health mitigation strategies (i.e. cancelling SXSW) and how has that changed over the next few weeks? Do you think we will look back after this pandemic and think we as a country: 1. Over reacted 2. Reacted appropriately 3. Under reacted?

Week 1, Group 3: Lessons Learned from History

- Compare and contrast two pandemics of your choosing. Highlight key differences in pandemic preparedness, responses, and outcomes.
- Based on your evaluation of prior pandemics, what are the biggest lessons learned and how can those be implemented into the current COVID-19 pandemic?

Week 1, Group 4: Public Health Communication

- Many people are buying N-95 masks for personal use because they heard these are the most protective. How would you design a public health campaign to educate the public about the use of N-95 masks and the need to allocate them to healthcare providers in high-risk situations (ie, risk of aerosolized virus)?
- You are a PCP at the VA in Austin. You want to create a public health messaging campaign about social distancing to reduce the risk of COVID-19 with a target audience of veterans >60yo who come to the VA.What information would you need to gather about your audience in order to create this campaign? How would you collect this information? How would you test your message? How would you tailor the same intended message as above for a population of young healthy adults at UT?
- Martin is a 67-year-old man with no PMH. Recently he saw a television commercial that reminds adults, especially those with health risks, to schedule an appointment for a flu shot. He thinks, “That doesn't apply to me, I’m in great health.” Later, while walking at the mall, he is confused after reading a poster that advises all adults, age 50 and over, to get a flu shot. He tells you that he heard coronavirus is no worse than the flu - and he’s never gotten that, so he should be fine. How would you explain to Martin why he might benefit from the flu shot? How would you tailor your message to Martin if he had high versus low health literacy? How would you assess that?
- 65 yo woman with progressive, metastatic cancer admitted to the hospital with fever, cough and dyspnea. She is being ruled out for COVID and on isolation in the ICU. Her husband is quite angry that it is taking nurses extra time to go in the room to attend to his wife’s needs because they have to put on PPE. He also thinks she needs more help breathing. He says to you, “You are taking too long. She rang her call button 10 minutes ago. You just don’t want to go in her room and get COVID. And I know that doctor isn’t going to put her on the breathing machine if she needs it because she has cancer and is too old.” What is the risk to nurses and all care providers of COVID-19 infection in this situation, and how to you communicate this risk to the patient? Should patients with advanced cancer get scare hospital resources in times of crisis? How would you talk to the patient’s husband about COVID, how to deal with fear, anger amongst family members? How do you talk to the nurses and other health care workers about care issues and support their fears and needs?

Week 2: Questions for entire class

- How has your country's respective culture played a role in their pandemic response? Discuss how social norms and mores, cultural traditions, language, religion, etc have factored into both the design and delivery of the pandemic response, and also to the public's acceptance and willingness to participate in or abide by pandemic response policies or mandates (or in some cases, their lack of acceptance and willingness!). What role do cultural institutions have in pandemic response, and/or the aftermath of the pandemic?
- In the countries / states / cities you're examining, what have you found to be major facilitators of a successful pandemic response? That is, what has allowed countries to respond quickly and effectively? Conversely, what have you found to be the major barriers to a successful pandemic response? What has held countries back or hampered progress? Share examples and references from the countries you're examining, and respond to each other to compare / contrast over this discussion thread. Perhaps even consider the question, "If you were the public health leader for your country/state/city, what would you have kept and what would you have done differently in the COVID-19 response?"
- Discuss some examples how the COVID-19 pandemic has exposed, or perpetuated, inequity in your respective countries. How is this playing out? What strategies are being taken to combat this? What lessons can different countries teach the other about how to prevent or address the exacerbation of social and health inequities during this pandemic?
- What aspects of your country's health policy framework played a major role in the design of their pandemic response? That is, what key policy considerations had to be accounted for or incorporated into the pandemic response? What policy-level factors either helped, or hindered, the response efforts? Similarly, what features of the design of your country's health care delivery system impacted the roll out of the pandemic response? You may want to consider thinking through the six WHO Health System Building Blocks (leadership/governance, financing, health care workforce, information management, supply chains, and service delivery) and analyzing how each of these played a role in the pandemic response and its effectiveness.
