# Supplementary material for: Global health on the front lines: an innovative medical student elective combining education and service during the COVID-19 pandemic
Source: BMC Med Educ. 2021 Mar 27;21:186. doi: 10.1186/s12909-021-02616-9 (PMC8003893; doi:10.1186/s12909-021-02616-9)
Supplement: Supplementary file 2 — Additional file 2. Pre-post questionnaires. [file 12909_2021_2616_MOESM2_ESM.docx]

**Global health on the front lines: An innovative medical student elective combining education and service during the COVID-19 pandemic**

**Authors and Affiliations:** Brandon S. A. Altillo, MD, MPH^1,2,3^, Megan Gray, MD, MPH^1,2^, Swati B. Avashia, MD^1,2,3^, Aliza Norwood, MD^1,3^, Elizabeth A. Nelson, MD^3,4^, Clarissa Johnston, MD^3,4^, Darlene Bhavnani, PhD, MPH^1^, Hemali Patel, MD^3^, Coburn H. Allen, MD^2^, Sarayu Adeni, MPA-DP^1^, Nicholas D. Phelps, PhD^1^, and Tim Mercer, MD, MPH^1,3^

^1^Department of Population Health, The University of Texas at Austin Dell Medical School, Austin, Texas, USA

^2^Department of Pediatrics, The University of Texas at Austin Dell Medical School, Austin, Texas, USA

^3^Department of Internal Medicine, The University of Texas at Austin Dell Medical School, Austin, Texas, USA

^4^Department of Medical Education, The University of Texas at Austin Dell Medical School, Austin, Texas, USA

**Corresponding Author:**

Dr. Tim Mercer, Department of Population Health, The University of Texas at Austin Dell Medical School, 1601 Trinity St., Bldg B., Austin, TX 78712, USA; telephone: 512-495-5393; email: [tim.mercer@austin.utexas.edu](mailto:tim.mercer@austin.utexas.edu).

**Additional File 2: COVID-19 Elective Pre-/Post-Course Questionnaire**

***Pre-Course Questionnaire***

1. Covid-19 can be defined as which of the following?

a. An endemic zoonosis present in many places and affecting many people and animals

b. An epidemic zoonosis which is sporadic in temporal and spatial distribution

**c. An emerging zoonosis which is newly appearing in a population**

2. Which receptor does SARS-CoV-2 bind to?

**a. ACE2**

b. CD4

c. Sialic acid

3. Which of the following specimen types is **NOT** currently used to test for SARS-CoV-2 infection?

a. Nasopharyngeal or oropharyngeal swabs

**b. Serum**

c. Aspirates of respiratory secretions

4. Which of the following agents in not currently being studied for the treatment of Covid-19?

a. Remdesivir

b. Favipiravir

c. Chloroquine

**d. Palivizumab**

5. According to the Institute of Medicine, what are the three CORE FUNCTIONS of public health?

a. Research, Vaccines, Surveillance

b. Research, Preventative Care, Policy Development

c. Preventative Care, Surveillance, Response

**d. Assessment, Policy Development, Assurance**

e. STIs, Vaccines, Create Panic

6. What is the third phase of a pandemic according to the WHO?

**a. Sporadic pockets of animal-human virus, no human to human transmission**

b. Widespread human to human transmission in multiple regions.

c. Past peak, still widespread disease.

d. Detection of novel animal virus with potential to infect humans

e. Zombie apocalypse

7. Which of the following tactics was utilized during the Influenza Pandemic of 1918?

a. Public funerals were prohibited, and countries involved in WWI implemented announcements regarding the outbreak.

b. Countries involved in WWI implemented announcements regarding the outbreak, and social distancing practices were eventually implemented.

**c. Public funerals were prohibited, and social distancing practices were eventually implemented.**

d. All of the above

8. Traditional public health measures used during SARS (2003) were successful and included active case detection, isolation of cases, contact tracing and quarantine of all contacts, social distancing, and community quarantine. Which of the following is **NOT** a reason that control methods for SARS may be less effective for COVID-19?

- 1. COVID-19 has a higher transmissibility than SARS.
  2. COVID-19 has a lower case-fatality rate than SARS.
  3. **It took longer to sequence the virus and establish a diagnostic assay for COVID-19 than for SARS.**
  4. Community spread happened more quickly and was more widespread with COVID-19 than for SARS.

9. Which of the following people would benefit from a payroll tax holiday, as proposed by the Trump administration to reduce the financial impact of the COVID-19 pandemic? **Select all that apply.**

1. A 19-year-old woman from Angola who is seeking asylum; she has no work permit yet but is cleaning houses for a couple of families to make ends meet
2. A 29-year-old musician who drives Uber and Lyft on weekends
3. **A 39-year-old man who makes $18 an hour in a short-term construction job**
4. A 49-year-old mother of 2 who works for a hotel; her hours have been cut to 0 but she has not been fired
5. A 59-year-old man who worked in a car factory but was laid off last week due to global supply chain disruption
6. **A 69-year-old woman who owns a food truck with 5 employees**
7. A 79-year-old retiree whose income and savings are primarily from investments

10. What are the three levels of health literacy?

a. Basic, informational, and higher-order

b. Informal, informational, and critical

**c.  Basic, communicative, and critical**

d.  Lay, communicative, and informational

11. Approximately what percentage of American adults have the highest level of health literacy?

a.      <5%

**b.      10-15%**

c.      25-30%

d.      40-45%

12. I feel confident in my ability to lead a conversation about limited PPE resources with clinical staff.

1. Strongly agree
2. Somewhat agree
3. Somewhat disagree
4. Strongly disagree

13. I feel confident in my ability to lead a conversation about limited ventilators with patients’ families.

1. Strongly agree
2. Somewhat agree
3. Somewhat disagree
4. Strongly disagree

14. I feel confident in my ability to address fear and anger with patients and families in the setting of COVID-19 infection.

1. Strongly agree
2. Somewhat agree
3. Somewhat disagree
4. Strongly disagree

15. What is your current level of anxiety or concern about the pandemic?

1. None
2. Minimal
3. Moderate
4. High

16. I have been meaningfully engaged in the local pandemic response.

1. Strongly agree
2. Somewhat agree
3. Somewhat disagree
4. Strongly disagree

17. Do you have previous experience in public health? If yes, what? (MPH, Peace Corps, etc)

1. Yes ________________
2. No

18. What do you hope to achieve by taking this course?

***Post-Course Questionnaire***

1. Covid-19 can be defined as which of the following?

a. An endemic zoonosis present in many places and affecting many people and animals

b. An epidemic zoonosis which is sporadic in temporal and spatial distribution

**c. An emerging zoonosis which is newly appearing in a population**

2. Which receptor does SARS-CoV-2 bind to?

**a. ACE2**

b. CD4

c. Sialic acid

3. Which of the following specimen types is not currently used to test for SARS-CoV-2 infection?

a. Nasopharyngeal or oropharyngeal swabs

**b. Serum**

c. Aspirates of respiratory secretions

4. Which of the following agents in not currently being studied for the treatment of Covid-19?

a. Remdesivir

b. Favipiravir

c. Chloroquine

**d. Palivizumab**

5. According to the Institute of Medicine, what are the three CORE FUNCTIONS of public health?

a. Research, Vaccines, Surveillance

b. Research, Preventative Care, Policy Development

c. Preventative Care, Surveillance, Response

**d. Assessment, Policy Development, Assurance**

e. STIs, Vaccines, Create Panic

6. What is the third phase of a pandemic according to the WHO?

**a. Sporadic pockets of animal-human virus, no human to human transmission**

b. Widespread human to human transmission in multiple regions.

c. Past peak, still widespread disease.

d. Detection of novel animal virus with potential to infect humans

e. Zombie apocalypse

77. Which of the following tactics was utilized during the Influenza Pandemic of 1918?

a. Public funerals were prohibited, and countries involved in WWI implemented announcements regarding the outbreak.

b. Countries involved in WWI implemented announcements regarding the outbreak, and social distancing practices were eventually implemented.

**c. Public funerals were prohibited, and social distancing practices were eventually implemented.**

d. All of the above

8. Traditional public health measures used during SARS (2003) were successful and included active case detection, isolation of cases, contact tracing and quarantine of all contacts, social distancing, and community quarantine. Which of the following is NOT a reason that control methods for SARS may be less effective for COVID-19?

1. COVID-19 has a higher transmissibility than SARS.
2. COVID-19 has a lower case-fatality rate than SARS.
3. **It took longer to sequence the virus and establish a diagnostic assay for COVID-19 than for SARS.**
4. Community spread happened more quickly and was more widespread with COVID-19 than for SARS.

9. Which of the following people would benefit from a payroll tax holiday, as proposed by the Trump administration to reduce the financial impact of the COVID-19 pandemic? **Select all that apply.**

1. A 19-year-old woman from Angola who is seeking asylum; she has no work permit yet but is cleaning houses for a couple of families to make ends meet
2. A 29-year-old musician who drives Uber and Lyft on weekends
3. **A 39-year-old man who makes $18 an hour in a short-term construction job**
4. A 49-year-old mother of 2 who works for a hotel; her hours have been cut to 0 but she has not been fired
5. A 59-year-old man who worked in a car factory but was laid off last week due to global supply chain disruption
6. **A 69-year-old woman who owns a food truck with 5 employees**
7. A 79-year-old retiree whose income and savings are primarily from investments

10. What are the three levels of health literacy?

a. Basic, informational, and higher-order

b. Informal, informational, and critical

**c.  Basic, communicative, and critical**

d.  Lay, communicative, and informational

11. Approximately what percentage of American adults have the highest level of health literacy?

a. <5%

**b.   10-15%**

c.   25-30%

d.   40-45%

12. I feel confident in my ability to lead a conversation about limited PPE resources with clinical staff.

1. Strongly agree
2. Somewhat agree
3. Somewhat disagree
4. Strongly disagree

13. I feel confident in my ability to lead a conversation about limited ventilators with patients’ families.

1. Strongly agree
2. Somewhat agree
3. Somewhat disagree
4. Strongly disagree

14. I feel confident in my ability to address fear and anger with patients and families in the setting of COVID-19 infection.

1. Strongly agree
2. Somewhat agree
3. Somewhat disagree
4. Strongly disagree

15. What is your current level of anxiety or concern about the pandemic?

1. None
2. Minimal
3. Moderate
4. High

16. I have been meaningfully engaged in the local pandemic response.

1. Strongly agree
2. Somewhat agree
3. Somewhat disagree
4. Strongly disagree

17. The service learning activities in the course contributed to my learning about pandemic response.

1. Strongly agree
2. Somewhat agree
3. Somewhat disagree
4. Strongly disagree

18. What was your level of knowledge about COVID-19 and pandemic response **BEFORE** participating in the activity?

1. None
2. Minimal
3. Moderate
4. High

19. What was your level of knowledge about COVID-19 and pandemic response **AFTER** participating in the activity?

1. None
2. Minimal
3. Moderate
4. High

20. How has this elective influenced your thinking about public health?

21. How has this elective impacted your sense of anxiety during this pandemic?
