# Supplementary material for: Global health on the front lines: an innovative medical student elective combining education and service during the COVID-19 pandemic
Source: BMC Med Educ. 2021 Mar 27;21:186. doi: 10.1186/s12909-021-02616-9 (PMC8003893; doi:10.1186/s12909-021-02616-9)
Supplement: Supplementary file 3 — Additional file 3. Institutional elective evaluation form. [file 12909_2021_2616_MOESM3_ESM.pdf]

Global health on the front lines: An innovative medical student elective combining education and service during the COVID-19 pandemic

Authors and Affiliations: Brandon S. A. Altillo, MD, MPH[1,2,3], Megan Gray, MD, MPH[1,2], Swati B. Avashia, MD[1,2,3], Aliza Norwood, MD[1,3], Elizabeth A. Nelson, MD[3,4], Clarissa Johnston, MD[3,4], Darlene Bhavnani, PhD, MPH[1], Hemali Patel, MD[3], Coburn H. Allen, MD[2], Sarayu Adeni, MPA-DP[1], Nicholas D. Phelps, PhD[1], and Tim Mercer, MD, MPH[1,3]

[1]Department of Population Health, The University of Texas at Austin Dell Medical School, Austin, Texas, USA

[2]Department of Pediatrics, The University of Texas at Austin Dell Medical School, Austin, Texas, USA

[3]Department of Internal Medicine, The University of Texas at Austin Dell Medical School, Austin, Texas, USA

[4]Department of Medical Education, The University of Texas at Austin Dell Medical School, Austin, Texas, USA

Corresponding Author:

Dr. Tim Mercer, Department of Population Health, The University of Texas at Austin Dell Medical School, 1601 Trinity St., Bldg B., Austin, TX 78712, USA; telephone: 512-495-5393; email: tim.mercer@austin.utexas.edu.

The University of Texas at Austin Dell Medical School	Dell Medical School Clinical	Evaluated By : evaluator's name<br>Evaluating : person (role) or moment's name (if applicable)<br>Dates : start date to end date

* indicates a mandatory response

Please provide feedback on various components of this Enrichment Elective. Thank you for participating so we can continuously improve these offerings.

General - please select your level of agreement with each statement below

	Strongly Disagree	Disagree	Neutral	Agree	Strongly Agree
*The learning objectives for this elective were clearly communicated.	○	○	○	○	○
*The elective activities/assignments helped me achieve the stated learning objectives.	○	○	○	○	○
*The elective was well organized.	○	○	○	○	○
*The requirements for passing the elective were clearly communicated.	○	○	○	○	○
*The elective promoted skills for self-directed/life-long learning.	○	○	○	○	○
*The elective promoted a positive environment for learning.	○	○	○	○	○
*I would recommend this elective to other students.	○	○	○	○	○

Overall - please select your level of agreement with each statement below

	Very Unsatisfactory	Unsatisfactory	Satisfactory	Very Good	Excellent
*Overall, the quality of this elective was . . .	○	○	○	○	○
*Overall, the quality of the teaching in this elective was . . .	○	○	○	○	○

Open Ended Questions - please write your comments or suggestions in the box provided

Why did you choose this elective?

What most helped you to accomplish your goals in this elective?

What could have improved your learning experience in this elective?

The following will be displayed on forms where feedback is enabled...
(for the evaluator to answer...)

*Did you meet with this student to provide face-to-face feedback?

- Yes
- No

(for the evaluee to answer...)

*Did you receive face-to-face feedback on your performance from a faculty member?

- Yes
- No

*Was the feedback sufficiently specific to guide your performance?

- Yes
- No
