# Supplementary material for: Global health on the front lines: an innovative medical student elective combining education and service during the COVID-19 pandemic
Source: BMC Med Educ. 2021 Mar 27;21:186. doi: 10.1186/s12909-021-02616-9 (PMC8003893; doi:10.1186/s12909-021-02616-9)
Supplement: Supplementary file 4 — Additional file 4. Focus group consent script and discussion guide. [file 12909_2021_2616_MOESM4_ESM.docx]

**Global health on the front lines: An innovative medical student elective combining education and service during the COVID-19 pandemic**

**Authors and Affiliations:** Brandon S. A. Altillo, MD, MPH^1,2,3^, Megan Gray, MD, MPH^1,2^, Swati B. Avashia, MD^1,2,3^, Aliza Norwood, MD^1,3^, Elizabeth A. Nelson, MD^3,4^, Clarissa Johnston, MD^3,4^, Darlene Bhavnani, PhD, MPH^1^, Hemali Patel, MD^3^, Coburn H. Allen, MD^2^, Sarayu Adeni, MPA-DP^1^, Nicholas D. Phelps, PhD^1^, and Tim Mercer, MD, MPH^1,3^

^1^Department of Population Health, The University of Texas at Austin Dell Medical School, Austin, Texas, USA

^2^Department of Pediatrics, The University of Texas at Austin Dell Medical School, Austin, Texas, USA

^3^Department of Internal Medicine, The University of Texas at Austin Dell Medical School, Austin, Texas, USA

^4^Department of Medical Education, The University of Texas at Austin Dell Medical School, Austin, Texas, USA

**Corresponding Author:**

Dr. Tim Mercer, Department of Population Health, The University of Texas at Austin Dell Medical School, 1601 Trinity St., Bldg B., Austin, TX 78712, USA; telephone: 512-495-5393; email: [tim.mercer@austin.utexas.edu](mailto:tim.mercer@austin.utexas.edu).

**Additional File 4: Focus Group Interview Guide**

***Focus Group Verbal Consent Script***

Hello, you have been selected to participate in a focus group discussing your experience in the COVID-19 elective. We hope that sharing your experience with other institutions will help increase capacity nationally and internationally for creating similar educational experiences for medical students.

There are minimal expected risks to participating in this study. You may experience discomfort or anxiety discussing issues related to an ongoing pandemic. If so, the Student Affairs office has counseling and other resources available. There is no compensation included in this study.

We will maintain all audio recordings in a secure password-protected drive, and only research staff will have access to audio records. No video recording will be saved. Information will be presented in summary format and you will not be identified in any publications or presentations; potentially identifiable quotes will be excluded.  Data from audio material may be used for future research studies, educational development, or conference presentations.

While the research team will protect data collected from this focus group to ensure your confidentiality, we cannot guarantee ​that other participants will not share what is said during this focus group. We encourage all of you to be respectful of each other’s confidentiality.

Your participation in this focus group is voluntary. You do not have to participate. If you choose not to participate, it will not affect your current or future participation or assessment in your medical school courses, including the COVID-19 elective. Your participation indicates your consent. If you do not wish to participate, you can log off from the conference call now.

***Focus Group Prompts***

- 1. What is it like to be a medical student during the COVID-19 pandemic?
  2. Are there any difficult questions you’ve gotten from family and friends about COVID-19?
  3. Do you feel confident discussing PPE shortages with staff?
  4. Do you feel confident discussing ventilator shortages with patients and families?
  5. What is your anxiety level about the COVID-19 pandemic?
  6. Has this changed as a result of this course?
  7. What has been helpful in dealing with anxiety?

1. What are your thoughts about your future in medicine?
2. Does the COVID-19 pandemic change your relationship with the field of medicine?
   1. How do you think the COVID-19 pandemic will affect your practice as a resident?
   2. Are you interested in medical leadership in a crisis?
3. What are some lessons already learned from the COVID-19 pandemic?
